# Efficient extracellular vesicle isolation by combining cell media modifications, ultrafiltration, and size-exclusion chromatography

**DOI:** 10.1371/journal.pone.0204276

**Published:** 2018-09-27

**Authors:** Eduarda M. Guerreiro, Beate Vestad, Lilly Alice Steffensen, Hans Christian D. Aass, Muhammad Saeed, Reidun Øvstebø, Daniela Elena Costea, Hilde Kanli Galtung, Tine M. Søland

**Affiliations:** 1 Institute of Oral Biology, Faculty of Dentistry, University of Oslo, Oslo, Norway; 2 Blood Cell Research Group, Department of Medical Biochemistry, Oslo University Hospital, Ullevål, Oslo, Norway; 3 Electron Microscopy Laboratory, Institute of Oral Biology, University of Oslo, Oslo, Norway; 4 Centre for Cancer Biomarkers CCBio and Gade Laboratory for Pathology, Department of Clinical Medicine, University of Bergen, Bergen, Norway; 5 Department of Pathology, Haukeland University Hospital, Bergen, Norway; 6 Department of Pathology, Oslo University Hospital, Oslo, Norway; University of South Alabama Mitchell Cancer Institute, UNITED STATES

## Abstract

Extracellular vesicles (EVs) are a heterogeneous population of biological particles released by cells. They represent an attractive source of potential biomarkers for early detection of diseases such as cancer. However, it is critical that sufficient amounts of EVs can be isolated and purified in a robust and reproducible manner. Several isolation methods that seem to produce distinct populations of vesicles exist, making data comparability difficult. While some methods induce cellular stress that may affect both the quantity and function of the EVs produced, others involve expensive reagents or equipment unavailable for many laboratories. Thus, there is a need for a standardized, feasible and cost-effective method for isolation of EVs from cell culture supernatants. Here we present the most common obstacles in the production and isolation of small EVs, and we suggest a combination of relatively simple strategies to avoid these. Three distinct cell lines were used (human oral squamous cell carcinoma (PE/CA-PJ49/E10)), pancreatic adenocarcinoma (BxPC3), and a human melanoma brain metastasis (H3). The addition of 1% exosome-depleted FBS to Advanced culture media enabled for reduced presence of contaminating bovine EVs while still ensuring an acceptable cell proliferation and low cellular stress. Cells were gradually adapted to these new media. Furthermore, using the Integra CELLine AD1000 culture flask we increased the number of cells and thereby EVs in 3D-culture. A combination of ultrafiltration with different molecular weight cut-offs and size-exclusion chromatography was further used for the isolation of a heterogeneous population of small EVs with low protein contamination. The EVs were characterized by nanoparticle tracking analysis, immunoaffinity capture, flow cytometry, Western blot and transmission electron microscopy. We successfully isolated a significant amount of small EVs compatible with exosomes from three distinct cell lines in order to demonstrate reproducibility with cell lines of different origin. The EVs were characterized as CD9 positive with a size between 60–140 nm. We conclude that this new combination of methods is a robust and improved strategy for the isolation of EVs, and in particular small EVs compatible with exosomes, from cell culture media without the use of specialized equipment such as an ultracentrifuge.

## Introduction

Extracellular vesicles (EVs) are a heterogeneous population of biological particles surrounded by a phospholipid membrane [1]. They have been classified as apoptotic bodies, microvesicles and exosomes, from the largest to the smallest [2], although the exact boundaries between subgroups remain unclear. Interestingly, they are present in all biological fluids and contain a myriad of biomolecules, such as proteins and nucleic acids. Thus, EVs are important players in cell to cell communication both in physiological and pathological conditions [3–6]. Therefore, they are a very attractive source of potential biomarkers for early detection of diseases such as cancer [7]. Indeed, tumor-associated EVs have been shown to be involved in the progression of cancer by modulating the microenvironment and even prime distant sites where metastasis may develop [8–10]. To better comprehend these vesicles and their specific roles, it is critical that EVs are isolated and purified in a robust and reproducible manner. A standardized method for EV isolation from cell culture supernatants, that is reproducible, practical and feasible for most laboratories, is currently lacking [11, 12]. Here we present known obstacles in the production and isolation of small EVs compatible with exosomes. We suggest a combination of relatively simple strategies to avoid these, adhering to the guidelines of the International Society for Extracellular Vesicles when possible [11].

EVs can be isolated from biofluids or from cell cultures [13]. In a methodological study such as the present, cell culture media is a convenient source of EVs as one can assure a high gain of vesicles from the same source in a reproducible manner [14]. Nonetheless, there are a few obstacles that must be taken into account. The use of fetal bovine serum (FBS) is such an example, as it is needed to promote cell growth, proliferation, and cell attachment. But it also contributes with great amounts of contaminating EVs [13, 15]. These serum EVs are very similar to the ones produced by the cultured cells, making it laborious to distinguish them during the isolation process [13, 16, 17]. Current practice is to remove FBS from the culture media shortly before EV collection [18]. However, this approach is likely to induce cell stress that negatively influences e.g. cell proliferation and viability, which in turn may influence the type, cargo, and amount of released EVs [19, 20]. As an alternative, the FBS can be replaced by an equal amount of exosome-depleted FBS; but this solution is quite costly [13, 21]. Another alternative is to step-wise reduce the FBS content to minimize cell stress.

Another obstacle in EV production is the constraint on the EV isolation yield that is due to the limited amount of cells that can be grown in classic cell culture flasks. Scaling up by increasing the number of flasks is an option, but is time consuming, costly, and produces high amounts of cell culture medium supernatant to further process. The Integra CELLine culture system (Argos Technologies, Vernon Hills, USA) can ameliorate these problems [22]. Due to its unique characteristics as a semi-continuous system, the Integra CELLine bypasses the classical flask limitations of limited oxygen supply and nutrient depletion [23, 24]. It is also reported that the CELLine system mimics physiological growth conditions by allowing 3D cell growth, which seems important for the dramatic increase in cell number while augmenting many-fold the amount of EVs recovered [22, 25].

In addition to the above mentioned issues concerning growth conditions, the current EV isolation methods all influence the final yield, purity, and/or concentration of the isolated vesicles [19]. The procedure most commonly used for the isolation of EVs has been ultracentrifugation (UC) where the samples are centrifuged at speeds of 100,000 or 120,000 g [26–28]. Although successful, this method can deform vesicles due to the high centrifugal forces [19]. In addition, UC will co-precipitate proteins, lipoproteins, and other contaminants [19]. Alternative approaches include immunoaffinity isolation methods, polymer-based precipitation techniques, or size-exclusion chromatography (SEC). While the first method targets specific membrane proteins, thus, isolating non-representative enriched subpopulations of EVs, the second precipitates not only the vesicles, but also a high amount of proteins by reducing the solubility of the EV suspension [11, 29]. Due to these concerns, the first two methods introduce significant disadvantages for downstream analysis of isolated EVs. In contrast, the last method, SEC, separates the components of a solution according to their size and allows for the separation of EVs from proteins, nucleic acids, and other biomolecules while maintaining their functional and morphological integrity [29, 30]. SEC is routinely used for the isolation of EVs from biological samples such as serum/plasma and saliva, where usually only small volumes of sample are available (from 0.5 μl up to 2 ml), still sufficient for downstream analyses [30–33]. On the other hand, the limited volume of sample that can be loaded onto the SEC columns is a significant drawback when isolating EVs from cell culture media. Here, vesicles are already much diluted, thus requiring large volumes to ensure a proper yield of EVs [19]. By using ultrafiltration (UF) prior to SEC, water molecules and smaller proteins will be forced through a porous membrane with molecular weight cutoffs (MWCOs) ranging from 3 to 100 kDa. This retains the EVs and the larger proteins in a reduced volume that can then be loaded onto a SEC column [34]. However, it is important to note that although the MWCO of the membrane affects the proteins that are retained, there is no information if there is any impact on the resultant EV population.

Due to the above mentioned concerns, we aimed to establish a robust and improved strategy for the isolation of EVs, and in particular small EVs compatible with exosomes, from cell culture media without the use of specialized equipment such as an ultracentrifuge. The yield of isolated EVs was improved by 1) optimizing the cell culture media through a slow adaptation to reduce the presence of contaminating EVs from FBS and diminish cell stress, 2) using culture flasks optimized for high-cellular density growth to increase the number of cells and thereby EVs in culture, and 3) combining UF and SEC for the isolation of a heterogeneous population of EVs compatible with exosomes from cell culture media.

## Materials and methods

### Cell culture

Commercially available cell lines from human oral squamous cell carcinoma (OSCC) (PE/CA-PJ49/E10) (ECACC, Salisbury, UK) and pancreatic adenocarcinoma (BxPC3, ATCC, Manassas, USA), in addition to a cell line from a human melanoma brain metastasis (H3; a kind gift from Prof. F. Thorsen, University of Bergen, Norway) were used [35]. The E10 cells were grown in Iscove's Modified Dulbecco's Medium (IMDM) (SIGMA), while BxPC3 cells were grown in Roswell Park Memorial Institute (RPMI) (Gibco, Life Technologies, Paisley, UK) medium. Both media were supplemented with 10% FBS (SIGMA), 2 mM L-glutamine (Thermo Scientific, Paisley, UK), and 1X Antibiotic Antimycotic Solution (penicillin, streptomycin, and amphotericin B (PSA), SIGMA), hereafter referred to as complete IMDM and complete RPMI. The H3 cells were cultured in Dulbecco’s Modified Eagle Medium (DMEM) (Gibco, Life Technologies) supplemented with 10% FBS, 2 mM L-glutamine, 1X PSA, and 4X non-essential amino acids (NEAA) (Gibco, Life Technologies, Bleiswijk, the Netherlands), further denoted as complete DMEM. Cells were kept at 37°C in a 5% CO_2_ atmosphere.

### Culture media optimization

#### FBS reduction and cell culture adaptation

**Fig 1:**
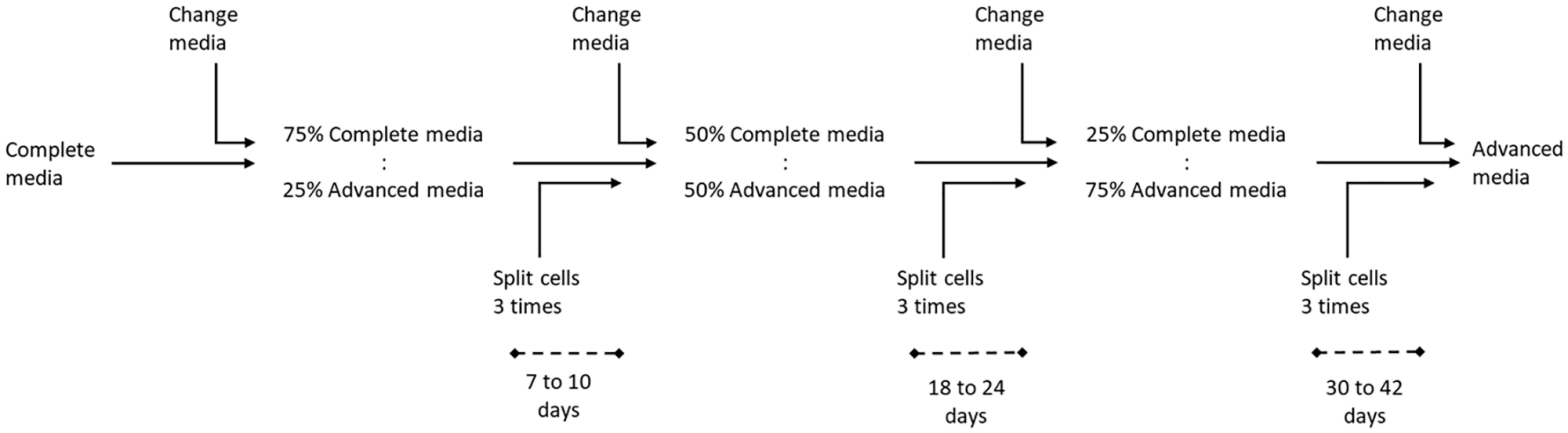
Flowchart for the adaptation of E10, BxPC3, and H3 cell lines to Advanced media. Cell adaptation was carried out by sub-culturing cells into stepwise increasing ratios of Advanced media to the conventional complete media until the conventional media were completely replaced. Cells were split 3 times between each change of culture media, when cells reached a confluence of approximately 80%. The time interval between the change of culture media increased from 7–10 days to 30–40 days as the percentage of the conventional media was decreased.

To avoid contamination by EVs from the FBS, the current practice is to remove this supplement from the media prior to collection. Thus, to determine the effect of this treatment, E10, BxPC3, and H3 cells were seeded in 6 well plates at 5x10^5^ cells/well, and grown in complete media. When cells reached 70 to 80% confluence, cell culture media were renewed (controls) or replaced with FBS-free media. Cells were incubated for 24 hrs at 37°C with 5% CO_2_. Since there are indications that such a sudden removal of FBS stresses cells, we investigated the effect of a gradual removal of FBS on cell proliferation. All cell lines were adapted to Advanced Reduced Serum Media (Advanced DMEM or Advanced RPMI) (Gibco, Life Technologies). These are culture media capable of supporting cell proliferation with a low amount of FBS due to their content of albumin (AlbuMAX II), transferrin (Human Transferrin; Holo), and insulin (Insulin Recombinant Full Chain), all important for cell survival (https://www.thermofisher.com/za/en/home/life-science/cell-culture/mammalian-cell-culture/classical-media/advanced-d-mem-and-mem.html) [36–38]. The media were supplemented with 2 mM L-glutamine and 1X PSA in addition to 1% exosome-depleted FBS (Gibco, Life Technologies) to minimize the presence of contaminating EVs. According to the producer, this FBS is ≥90% exosome-depleted. Briefly, cell adaptation was carried out by sub-culturing cells from the conventional complete media into stepwise increasing ratios of Advanced media (75%:25%, 50%:50%, 25%:75%) until the conventional media were completely replaced with Advanced media. A flowchart of the cell adaptation from complete to Advanced media is presented in Fig 1. Thereafter, sub-culturing was carried out in Advanced DMEM (E10 and H3) or Advanced RPMI (BxPC3) for 8 weeks for the E10 and BxPC3 cell lines and for 10 weeks for the H3 cell line. Although E10 cells were normally grown in IMDM, no Advanced IMDM was commercially available, thus this cell line was adapted to the Advanced DMEM. In addition, cells were also adapted to conventional media with 1% FBS, conventional media with 1% exosome depleted FBS and Advanced media with 1% FBS.

We investigated whether changes in the various growth media influenced cell proliferation. Cells grown in 1) conventional media with 10% FBS, and cells fully adapted to 2) conventional media with 1% FBS, 3) conventional media with 1% exosome depleted FBS (E10 only), 4) Advanced media with 1% FBS, and 5) Advanced media with 1% exosome depleted FBS were seeded into 6 well plates, 2.0x10^5^ (E10 and BxPC3) or 1.0x10^5^ (H3) cells per well, in duplicate. Cells were collected by trypsination every 24 hrs for a total of 6 days and counted on a MoxiZ Mini Automated cell counter (Orflo Technologies, USA). The effect of the various culture media adaptations on cell proliferation was compared.

### Isolation of EVs

The E10, BxPC3, and H3 cell lines fully adapted to the Advanced media supplemented with 1% exosome-depleted were used for the isolation of the EVs (see below).

#### Collection of EV-enriched supernatant

**Fig 2:**
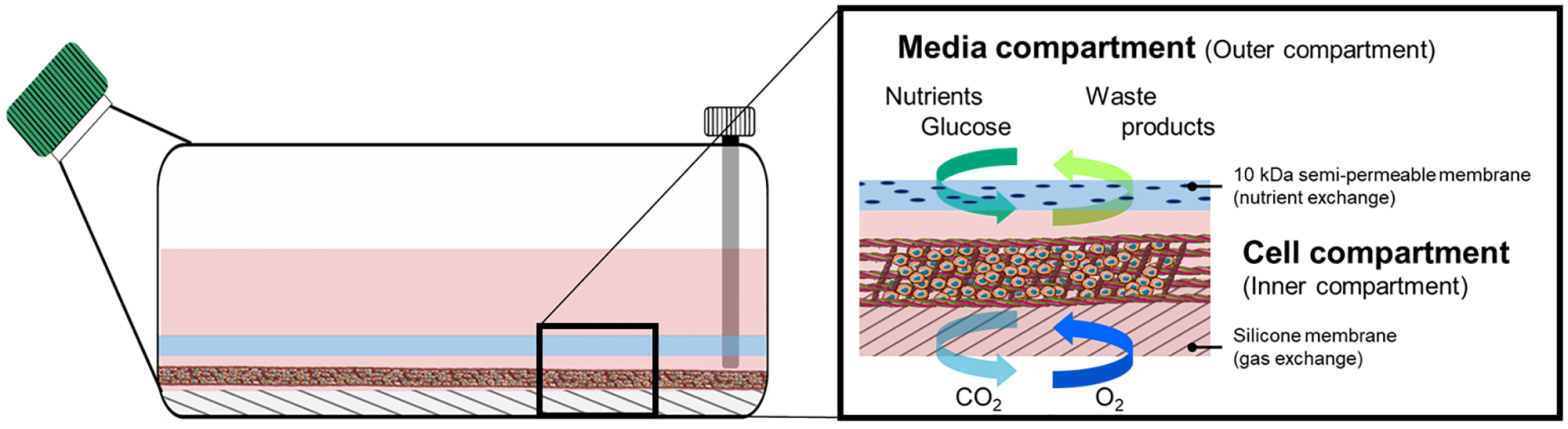
Schematic illustration of the CELLine AD1000 flask. This is a two-compartment culture system with an inner (cell) compartment design to sustain cell growth at high densities and an outer (media) compartment where cell free culture media is placed. Here, the outer and inner compartments are separated by a semi-permeable membrane which allows a continuous exchange of nutrients and waste. A woven mesh inside the cell compartment provides cells with support for adherence and growth. A silicone membrane at the bottom allows for direct oxygenation and gas exchange. Each compartment is accessed by a specific port. The medium reservoir is reached through the green cap and the inner, cell compartment through the white cap.

The E10, BxPC3, and H3 cell lines were cultured in the CELLine AD1000 (Argos Technologies, Vernon Hills, USA), a two-compartment culture flask where the inner compartment is specific for cell culturing while the outer chamber is for introducing cell-free culture media. These two compartments are separated by a semi-permeable cellulose acetate membrane with 10kDa pores that allows continuous nutrient diffusion and waste elimination. Within the inner compartment, a woven polyethylene terephthalate (PET) mesh provides a 3D-structure for cell growth. The bottom silicone membrane of the inner compartment provides direct oxygenation and gas exchange. Two ports give separate access to these compartments (Fig 2).

Briefly, 25x10^6^ cells resuspended in 16 ml of culture media were seeded in the inner compartment of the flasks and 500 ml of media was added to the outer compartment. The culture medium in the inner compartment was collected and replaced weekly. At the same time point, the medium from the outer compartment was collected and discarded. Fresh culture media was then added. Cells were incubated at 37°C with 5% CO_2_. The CELLine system increased the EV outcome 10-fold as shown by nanoparticle tracking analysis (NTA) and Western blot (WB) targeting CD9, see below; compared to the conventional T175 flask (data not shown). Additionally, to inspect the cell growth within the CELLine reactor, we performed scanning electron microscopy (SEM) of the interior of the flask. Briefly, 4% glutaraldehyde solution was added to the empty cell compartment and incubated at 4°C for 48 hours. Then dehydration was carried out by washing the inner compartment with milliQ water followed by 15 min incubations in an increasing series of ethanol dilutions (30%, 50%, 70%, 90%, 100% and 100%), and a final 1 hr incubation with 100% ethanol. Furthermore, the reactor flask was dismantled and all the elements from the cell compartment (the top semi-permeable cellulose acetate membrane, the inner woven PET and the bottom silicone membrane) were sputtered with a thin layer of gold. These reactor components were then observed in a Philips XL30 ESEM (Philips, FEI, Netherlands) operated at 12 kV.

The CELLine system was used throughout the remainder of the study, as this demonstrated a successful EV production and isolation (see Results). Approximately 18 ml of cell culture supernatants were collected (as described above) from each CELLine flask every week. Simultaneously, 20 ml of PBS was first added to the inner cell compartment for washing and then collected. Supernatants and PBS were pooled and stored at -80°C until a total of 50 ml of sample were collected (1 replicate). A total of 5 replicates per cell line were prepared.

#### EV isolation and purification by size-exclusion chromatography (SEC)

The samples described under “Isolation of EVs” were thawed and centrifuged in a Megafuge 1.0R (Heraeus Instruments) at 4000g for 5 min to discard cell debris. Supernatants were then centrifuged for 45 min at 15000g, 20°C in a fixed rotor Centrifuge 5804R (Eppendorf) to remove larger vesicles. Furthermore, the supernatants from the 15000g centrifugation were concentrated by ultrafiltration (UF) using Amicon-Ultra 15 Centrifugal Filter Units (Merck Millipore, Tullagreen, Cork, Ireland) with 3 different molecular weight cut-offs (MWCO): 30 kDa, 50 kDa, and 100 kDa. Briefly, 15 ml of the supernatants were loaded into each of the Amicon-Ultra 15 Centrifugal Filter Units (Ultracel-30, Ultracel-50, and Ultracel-100). Filter units were centrifuged for 30 min in a Megafuge 1.0R (Heraeus Instruments) equipped with a swing rotor to reduce the volume to 4 ml.

SEC columns were prepared as described in Supporting information (S1 Text). A pilot indicated that upon concentration, the CELLine samples became viscous, impairing a good separation of the EV-enriched fractions during SEC. Therefore, SEC columns were scaled up from 10 to 30 ml, thus requiring less concentrated sample (4 ml instead of 1 ml) to be used.

The 30 ml sepharose CL-2B (GE Healthcare Bio-Sciences AB, Uppsala, Sweden) SEC columns were washed once with 60 ml of filtered PBS (Millex-VV Syringe Filter unit, 0.1 μm, Merck Millipore). Thereafter, the concentrated samples from above (4 ml) were loaded onto the columns. After the whole sample had entered the column matrix, filtered PBS was continuously added in order to ensure a complete drainage of the sample. The eluate was collected by gravity in 30 sequential fractions of 1 ml. For each fraction, the amount of protein was determined by spectrophotometry (Absorbance 280nm, Nanodrop, Thermo Fisher Scientific).

### Characterization of EVs

The first three protein-enriched SEC fractions were used for EV characterization by nanoparticle tracking analysis (NTA), flow cytometry, Western blot (WB), and transmission electron microscopy (TEM) (see below).

#### Nanoparticle tracking analysis

Particle concentration and size distribution were obtained as previously described [28]. Briefly, analyses were carried out on a NanoSight NS500 instrument (Malvern Instruments, Amesbury, UK) equipped with a 488 nm laser, a high sensitivity sCMOS camera, and a syringe pump. Samples described above were diluted 20–50 times in 0.02 μm filtered PBS to obtain a concentration within the range of 10^8^−10^9^ particles/ml. Analysis was carried out with the NTA software (version 3.1 Build 3.1.54) using 60 seconds of video captures per sample (in triplicate) with a syringe pump speed of 20. Camera level was set to 14 and detection threshold was set to 3.

#### Immunoaffinity capture and flow cytometry

Immunoaffinity capture (IAC) of CD9^+^ EVs from the samples described above was carried out using the Exosome Human CD9 Flow Detection Kit (Dynal, Thermo-Fisher Scientific) followed by flow cytometry detection [39]. Specifically, 100 μl of the samples were incubated with 20 μl of prewashed anti-CD9 coated Dynabeads (2.7 μm) was carried out on a Testtube rotator mixer at 4°C overnight. The bead-bound EVs were then washed three times with 0.1% bovine serum albumin (BSA) in 0.1 μm filtered PBS and incubated with Anti-human CD9-RPE clone ML-13 (BD Biosciences, Oslo, Norway) or isotype control (IgG1-RPE, BD Biosciences) for 45 minutes at room temperature in an orbital shaker (1000 rpm) while protected from light. Samples were washed twice with 0.1% BSA in PBS prior to flow cytometry analysis using a BD FACS Aria Cytometer (BD Biosciences). Median fluorescence intensity (MFI) was reported as a signal to noise (S/N) ratio to isotype control from a total of 3000 singlet events.

#### Detection of EV biomarker by Western blot

Western blot for the vesicle biomarker CD9 was performed on the samples described under “Isolation of EVs”. Here, 20 μl of each sample was loaded onto a 10–20% CriterionTM TGX^™^ polyacrylamide gel (BioRad). Following electrophoresis, proteins were transferred to a nitrocellulose membrane (0.45 μm, BioRad). Then, membranes were blocked for 1 hr at room temperature in 5% milk in TBST (Tris-buffered saline, 0.1% Tween 20) and incubated with mouse anti-CD9 antibody (10626D, Invitrogen, Carlsbad, USA) in a dilution 1:1000, overnight at 4°C. Next, membranes were washed and incubated with the secondary HRP-conjugated antibody anti-mouse IgG, horseradish peroxidase linked (NA9310V, GE Healthcare, UK, 1:10000) for 1 hr at room temperature. Band detection was carried out using SuperSignalTM West Dura Extended Duration Substrate (Thermo Scientific, Rockford, USA) in a ChemiDoc Touch Imaging System (BioRad).

#### Transmission electron microscopy (TEM)

For TEM analysis, 100 mesh Formvar/Carbon coated copper grids (Electron Microscopy Sciences, Hatfield, USA) were placed on top of 15 μl droplets of the samples described above in “EV isolation and purification by size-exclusion chromatography (SEC)”. Following incubation for 5 min at room temperature, the grids were washed 3X with milliQ water. The preparations were further transferred to a drop of a 4% (w/V) uranyl acetate solution (aqueous) and incubated for 1 min for negative staining. Grids were then examined in a Philips CM120 BioTwin transmission electron microscope.

## Results

### Cell proliferation studies indicate that Advanced 1% exosome-depleted FBS medium is the best compromise to reduce contaminating EVs from FBS

**Fig 3:**
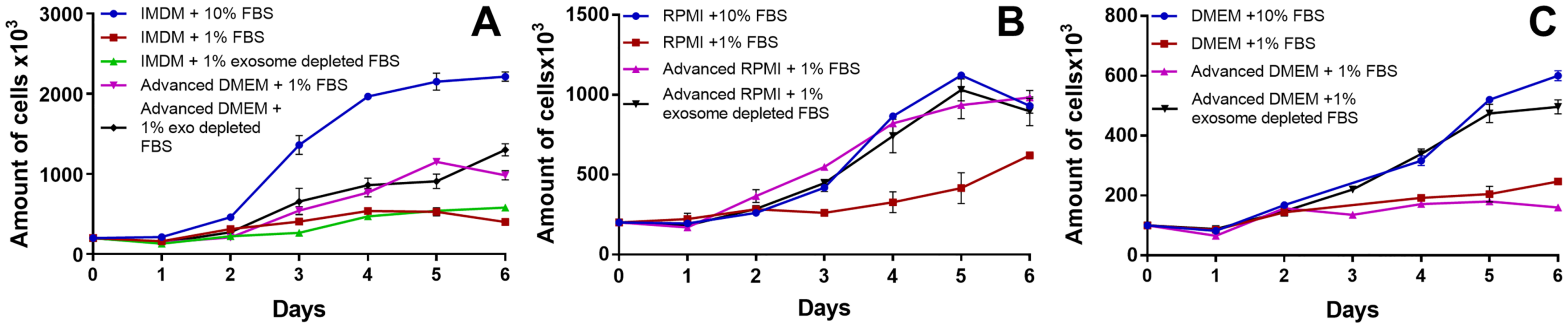
Growth curves for E10 (A), BxPC3 (B), and H3 (C) cell lines fully adapted to the different culture media. Cells were either grown in conventional media with 10% FBS (blue line), conventional media with 1% FBS (red line), conventional media with 1% exosome depleted FBS (green line, E10 only as the BxPC3 and H3 cell lines were unable to proliferate in these conditions), Advanced media with 1% FBS (purple line), or Advanced media with 1% exosome depleted FBS (black line). The best growth media with the least vesicles contamination was Advanced media supplemented with 1% exosome depleted FBS (black line).

Growth curves indicating cell numbers in conventional media with 10% FBS and cells grown in the other media are compared in Fig 3. Reducing the regular FBS from 10% (blue curves) to 1% (red curves) in conventional media greatly reduced cell numbers. However, advanced media supplemented with 1% regular FBS (purple curves) or 1% exosome-depleted FBS (black curves) showed an improved proliferation. Although there is a reduction in proliferation in the new media, the cells still grow better than those in the conventional media supplemented with 1% FBS. Since we wished to reduce the amount of contaminating EVs, we selected the Advanced media with 1% exosome depleted FBS for the remainder of the study.

### The CELLine reactor ensures 3D-cell growth and an increased EV yield

**Fig 4:**
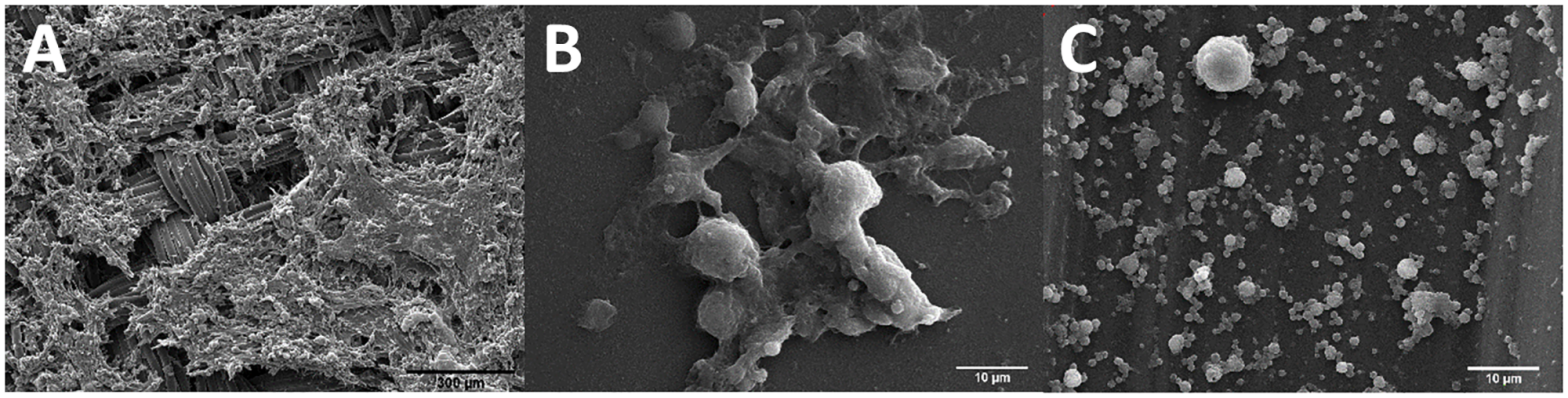
Scanning electron microscopy images of the interior of the CELLine reactor containing E10 cells. A: 3D cell growth in the mesh membrane of the inner compartment. Of interest, cell growth was also demonstrated in B: Interior aspect of the membrane separating the inner and outer compartments and C: Silicone bottom membrane, below the mesh membrane.

As indicated in a pilot, the CELLine system increased the EV outcome 10-fold compared to the conventional T175 flask as measured by NTA analysis and WB targeting the EV marker CD9 (data not shown). An inspection of the interior of the CELLine reactor demonstrated the growth of cells in a 3D pattern on the mesh membrane within the inner, cell compartment (Fig 4A). Cell growth was also demonstrated on the membrane separating the inner and outer compartments, on the surface facing the inner compartment (Fig 4B) and the bottom of the flask, below the bottom mesh membrane (Fig 4C).

### Isolation and characterization of EVs

#### Protein quantification

Collectively, protein quantification of all 30 SEC fractions revealed a similar peak for all cells lines and MWCOs, beginning in fraction 10 for E10 and BxPC3 and fraction 9 for H3 (see S1 Fig). As expected, this was not observed for the cell culture media (blanks). The protein concentrations in the BxPC3 fractions were about 2.5 times less than for the other two cell lines. The protein concentrations increased gradually from fraction 10–12 for E10 and BxPC3 and fraction 9–11 for H3. Therefore, these fractions were used for further EV characterization. The first 3 out of the 5 replicates for each cell line and each MWCO were further used (see section “Characterization of EVs” above). In addition, the different MWCO of the UF devices used to concentrate the samples prior to SEC did not influence the amount of proteins in these fractions.

#### Nanoparticle tracking analysis

**Fig 5:**
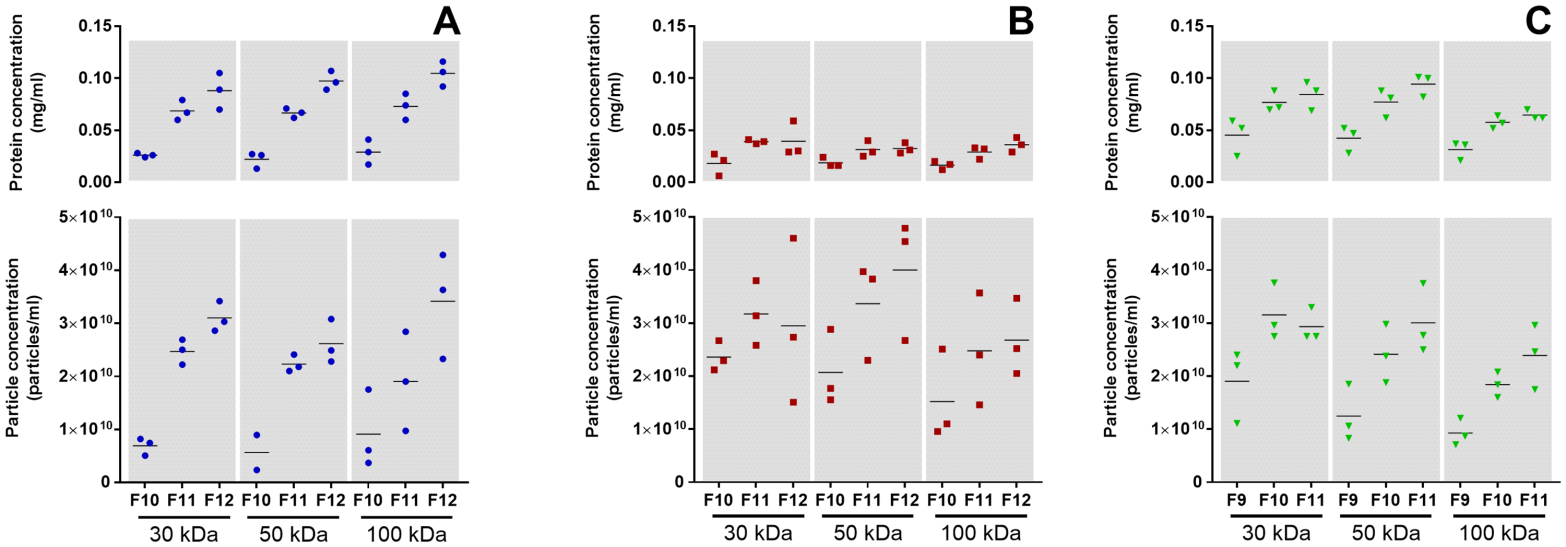
Protein and particle concentrations in EV-enriched fractions. The EV protein and particle concentrations from cell lines E10 (A), BxPC3 (B), and H3 (C) follow each other in a parallel manner within each molecular weight cutoff (MWCO) (n = 3).

Particle and protein concentrations were found to follow each other, and this was observed within each molecular weight cut off (MWCO) (Fig 5). The lowest particle concentration was found in E10 (Fig 5A), while the highest concentration was found in BxPC3 (Fig 5B), followed by H3 (Fig 5C). EV-enriched fractions from the E10 and H3 cell lines had a higher protein concentration than the corresponding fractions from BxPC3 (Fig 5).

#### CD9 detection by flow cytometry and Western blot

**Fig 6:**
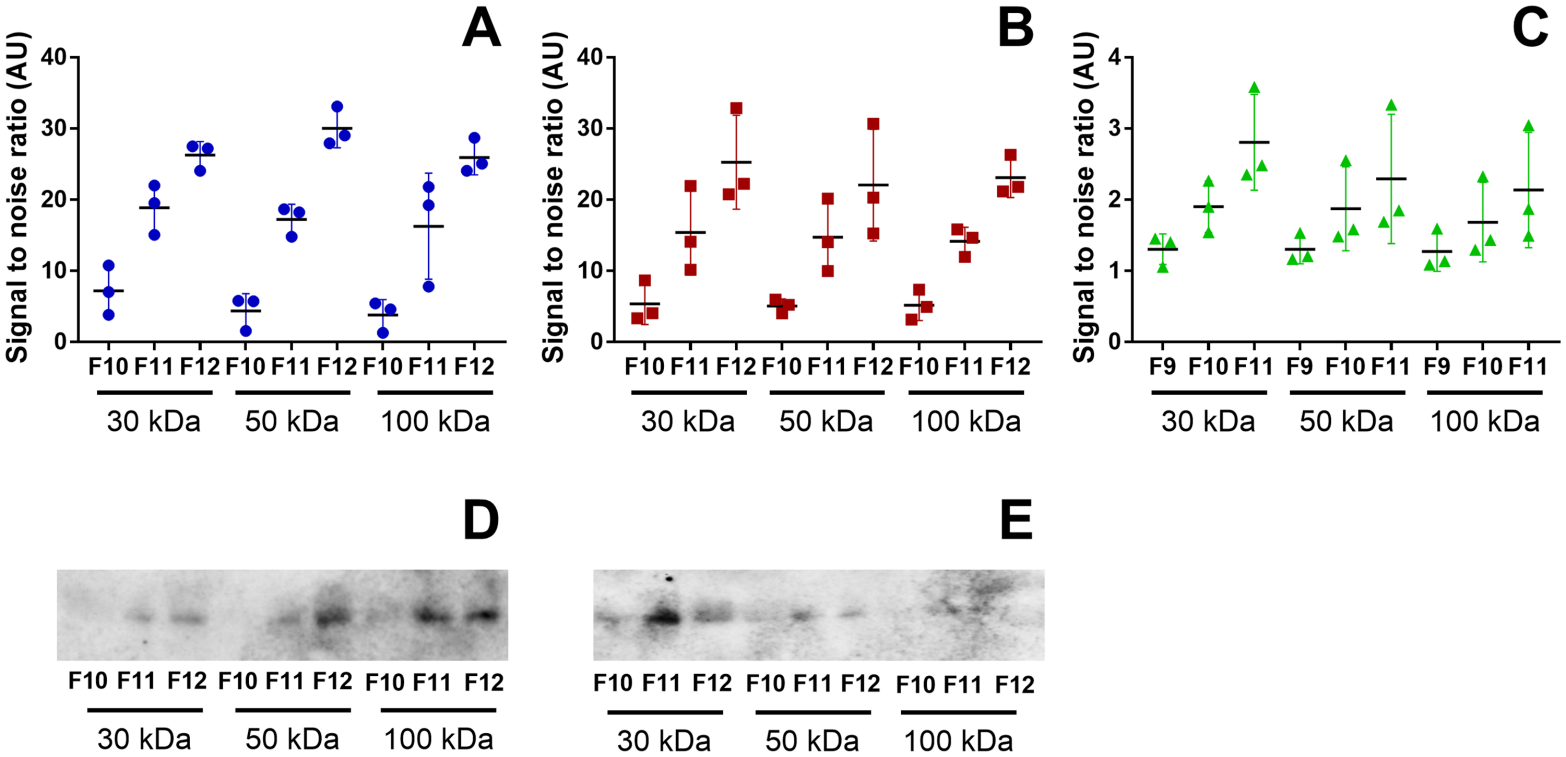
Flow cytometry and WB analysis targeting the exosome marker CD9 on isolated vesicles. CD9 positive vesicles were detected by flow cytometry. Median fluorescence intensity (MFI) was reported as a signal to noise (S/N) ratio to isotype control in EVs isolated from E10 (A), BxPC3 (B) and H3 (C) cells (n = 3). The presence of CD9 was also analyzed by WB, which was detected in vesicles from E10 (D) and BxPC3 cells (E) (n = 3).

Analysis of the classical exosome marker CD9 was performed by flow cytometry and WB. Results from flow cytometry showed a higher signal in vesicles from E10 (Fig 6A) and BxPC3 (Fig 6B) than for vesicles from the H3 cell line (Fig 6C). Nevertheless, for all cell lines and for all MWCOs, the first fractions presented the lowest signal, increasing towards the third fraction. The CD9 positive vesicles were also detected by WB for E10 (Fig 6D) and BxPC3 (Fig 6E). In contrast, no signal was detected in vesicles from H3 (data not shown).

#### Transmission electron microscopy

**Fig 7:**
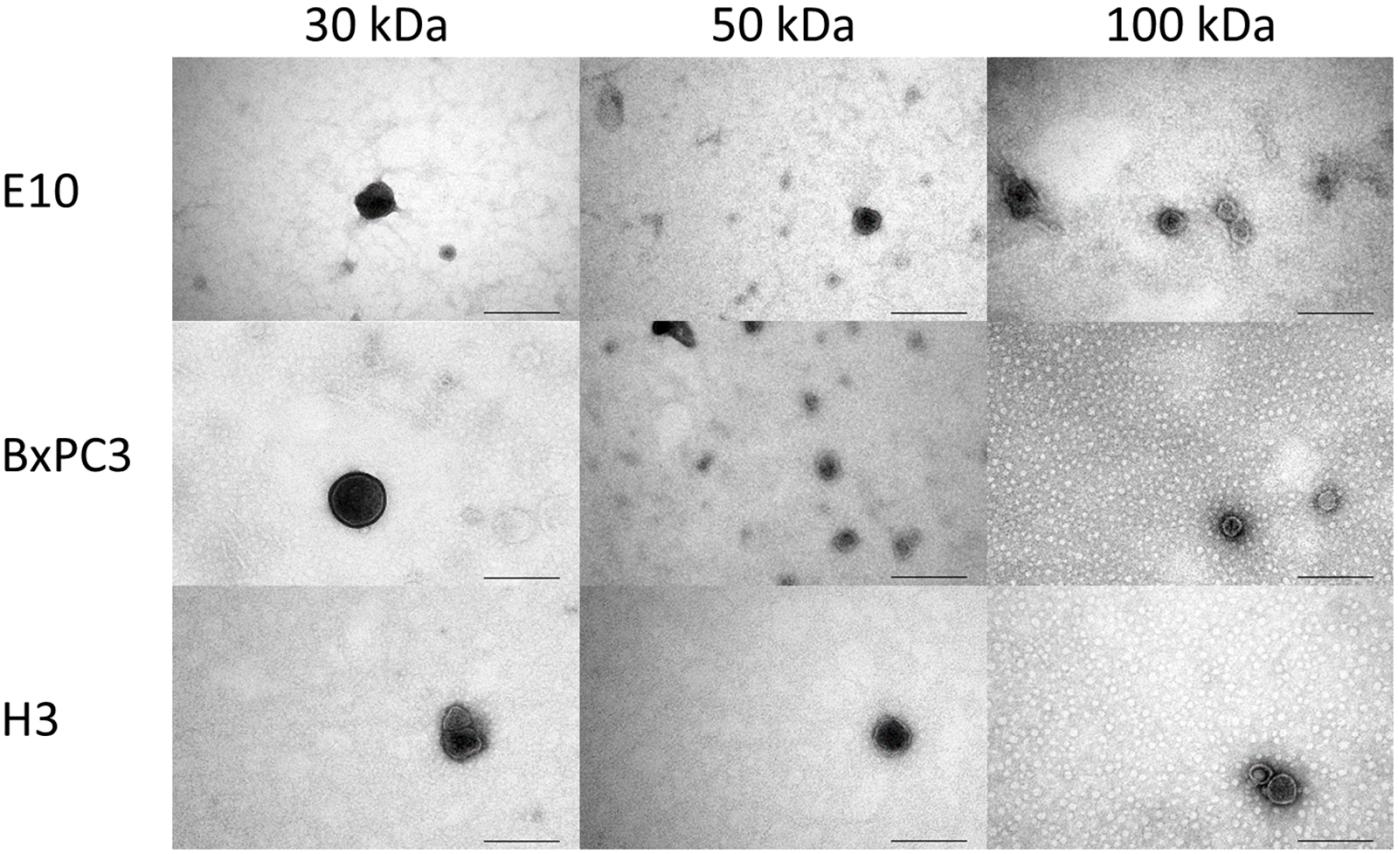
Transmission electron microscopy (TEM). Representative images from TEM of isolated vesicles from E10, BxPC3 and H3 cell lines using ultrafiltration devices with MWCO at 30, 50, and 100 kDa. Scale bars: 200 nm.

Characterization of isolated EVs was also performed with TEM (Fig 7). Observations using TEM showed the successful isolation of small EVs with a dark central area surrounded by a lighter peripheral zone, with diameters in the range of 60–140 nm, all characteristic features of exosomes [18, 40]. The MWCO did not appear to have an effect on particle size or morphology.

## Discussion

In this study we present a simple, robust, and reliable method for the isolation of EVs from cell culture supernatants. We used three different cancer cell lines representing malignancies of epithelial and mesenchymal origin. We first optimized the cell culture conditions by modifying the culture media to reduce the presence of contaminating EVs from serum supplements without adversely influencing cell proliferation. In addition, cell density in culture and EV yield was increased by using the CELLine culture flasks. Moreover, by incorporating ultrafiltration (UF) in combination with size-exclusion chromatography (SEC), we recovered EVs from three distinct cancer cell lines in a consistent manner, as determined by nanoparticle tracking assay (NTA), flow cytometry, Western blot (WB), and electron microscopy (EM).

### Exosome-reduced 1% FBS combined with advanced media is essential for successful cell growth and a high EV yield

As serum EVs mix with the ones produced by the cells in culture, it is time consuming and difficult to distinguish and separate the two. Thus, in most studies, the culture media are replaced with FBS-depleted media in one single step, known as starvation, when cultures are at 70–80% confluence. However, this sudden modification of the culture conditions is known to trigger several negative cellular responses [20, 41]. The effects of the removal of FBS can be observed at the level of cell proliferation, which can slow down or even stop, and at cell death level that may increase. Furthermore, starvation may also influence protein expression [20, 41], and EVs as they are, to a certain extent, mirrors of the cells that release them [1, 42]. Thus, a cellular stress response such as starvation might trigger changes in the cellular metabolism and synthesizing activities, thereby altering the production of EVs and their content [43, 44]. In order to limit acute stress due to starvation, we gradually adapted cells to exosome-depleted 1% FBS as a part of our pilot experiments. In this manner, the cell culture media were still supplemented with some serum while the presence of contaminating vesicles was reduced by more than 90% in comparison with the standard FBS. However, simply adding exosome-depleted FBS in the conventional culture media negatively affected cell proliferation (Fig 3A, green curve). This problem was overcome by combining the exosome-depleted FBS with Advanced culture media, which are designed to support cell proliferation in a low amount of FBS due to their content of albumin, transferrin, and insulin, all important for cell survival [36–38]. For the BxPC3 and H3 cell lines, this new culture condition did not adversely affect cell proliferation (Fig 3B and 3C, black curves; compared to conventional medium with 10% FBS, blue curves). For the E10 cells, on the other hand, proliferation slightly decreased in comparison with control (Fig 3A, black curve). Nevertheless, the combination of Advanced DMEM with 1% exosome-depleted FBS out-performed the other media tested while providing the least amount of contaminating EVs. Therefore, we consider the Advanced media supplemented with 1% exosome depleted FBS to be a good compromise between a minimal contamination with bovine vesicles and the negative effects on cell proliferation.

Moreover, standard culture T-flasks have limited surfaces where adherent cells can proliferate and grow, thus, restricting the final yield of EVs. To overcome this problem, Mitchell *et al*. used the CELLine culture system and successfully increased the amount of isolated EVs [22]. In their study, these EVs were comparable in phenotype, morphology and immune modulatory functions to the ones isolated from standard conditions. In our pilot with E10 cells, NTA showed a 10-fold increase in particle concentration in the supernatant from the CELLine flasks when compared to the conventional T175 flasks (data not shown). Possibly, this could be due to the 3D-growth environment, however, this is not known. Furthermore, the great amount of cells that is possible to culture in the CELLine flask requires much less space in the incubator, demands little handling (i.e., trypsination, centrifugation, reseeding), and produces a reduced volume of culture media to be processed. Therefore, we carried out the rest of the study using the CELLine culture flasks.

### Ultrafiltration followed by SEC allows for robust EV isolation

There are different methods available for isolation of EVs. Sizes and/or surface markers of EVs may vary depending on the isolation method used. This makes data comparability difficult [12, 45, 46]. The most common isolation approach has been ultracentrifugation (UC), which can be time consuming, operator dependent and cause vesicle damage [19, 29]. Additionally, this method requires the use of equipment that is not always commonly available. The use of magnetic beads or precipitation methods is less demanding in terms of equipment and are, thus, more practical [29]. However, the first method will isolate specifically enriched subpopulations of EVs, while the second precipitates heterogeneous populations of EVs together with contaminating proteins, and can even lead to vesicle damage [11, 29, 47]. SEC has been described as a third approach for EV isolation, separating them from proteins and other biomolecules, while maintaining vesicle integrity and function [47, 48]. For these reasons we decided to adopt SEC in our study. The work from Böing *et al*. describes the application of 10 ml sepharose CL-2B columns for the efficient isolation of EVs from small volumes of plasma [49]. While plasma is rich in vesicles, culture media have a comparatively low concentration of EVs. Thus, a vast volume of culture media is needed in order to produce a sufficient amount of vesicles [19].

First we ensured that the volume of media was compatible with the SEC columns. We used UF devices to concentrate the culture media in order to enrich the vesicle concentration. Initially, we performed a pilot concentrating the cell culture supernatant from the E10 cell line using UF devices with a 50 kDa MWCO. The samples were concentrated to 1 ml to be compatible with the 10 ml SEC columns. However, when thus concentrated, the CELLine samples became very viscous and, therefore, troublesome to elute, which impaired separation (data not shown). To overcome this challenge, we scaled up the SEC columns to 30 ml by increasing the cross-sectional area and the bed height (S1 Text) [29]. In this manner, it was possible to increase the sample volume, thus circumventing the viscosity problem. Before SEC in the 30 ml columns, ultrafiltration was carried out using 30, 50, and 100 kDa MWCO.

### Characterization of isolated EVs by protein quantification, NTA, WB, immunoaffinity capture, flow cytometry and EM

To ensure the successful isolation of EVs, and in particular the small EVs compatible with exosomes, sample characterization was carried out by protein quantification, NTA, WB, flow cytometry, and EM. Protein quantification of all SEC fractions was performed, and an early peak, beginning in fractions 9 or 10, was present for all the cell culture supernatants. These early peaks in protein concentration were due to the EVs eluting first through the SEC column. As the EVs are larger than the beads pores of the SEC columns, they cannot enter the sepharose gel particles. Thus, compared to proteins, they have less column volume to traverse and will elute first [29, 50].

Interestingly, Benedikter *et al*. used UF with the very stringent 10 kDa MWCO followed by SEC to successfully isolate EVs and proteins in culture media [51]. In the present study we tested 30, 50, and 100 kDa MWCOs. We found that the different MWCOs of the UF devices used to concentrate the samples prior to SEC did not influence the amount of proteins in the EV fractions. As the UF devices discriminate the proteins according to their MWCO, they will allow the smaller proteins to flow through, removing them from the concentrated culture media loaded into the SEC column. Therefore, influence of the different MWCOs was only found in the protein concentrations of the later fractions, where the soluble proteins are found.

Analysis by NTA showed, independently of cell line and MWCOs, an increase in particle concentration from the first to the third SEC fractions selected that accompanied a similar increase in protein concentrations (Fig 5). These results highlight that protein quantification is a good preliminary assessment to narrow down the EV-enriched SEC fractions from cell culture supernatants. However, the protein concentration does not necessarily mirror the amount of particles. This is the case for EVs isolated from BxPC3 cells. Here, despite a lower protein concentration of SEC fractions, the amount of particles was similar or slightly higher than for the other two cell lines.

Further characterization of the isolated vesicles was performed by flow cytometry and WB using the classical exosome marker CD9. Flow cytometry showed an increase of CD9 positive EVs from the first to the third fractions (Fig 6), similar to what was observed in protein and particle concentrations, for all cell lines and all MWCOs. However, there were less CD9 positive EVs from H3 cells (Fig 6C) than for E10 (Fig 6A) or BxPC3 cells (Fig 6B). To an extent, WB results reflected these results, as CD9 was detected in EVs from E10 (Fig 6D) and BxPC3 cells (Fig 6E), but not from H3 cells. This can be explained by the fact that WB is a much less sensitive detection method than flow cytometry. Other possible EV markers selected from the literature (TSG 101, Alix, CD63, and CD81) were tested by WB, but no signal was detected (data not shown). Either, the amount of markers was below the detection limit for WB, or the EVs isolated by the present method do not express these particular proteins. Nonetheless, TEM analysis confirmed the presence of vesicles with expected size and a double membrane compatible with exosomes for all cell lines and MWCOs in our study (Fig 7).

Since the aim of this study was to present a simple and robust EV isolation method, a further analysis of cytosolic, intracellular, or extracellular proteins was beyond our scope. However, such scrutinization will be performed in future studies.

In conclusion, modifying the conventional cell culture media to Advanced media in combination with 1% exosome depleted FBS made it possible to limit the presence of contaminating EVs while still ensuring an acceptable cell proliferation. The use of the CELLine reactor allowed a high cellular density in 3D-culture, thus increasing the amount of EVs produced. The characterization of the SEC fractions from the different cell lines by NTA, immunoaffinity capture, flow cytometry, WB and TEM validated successful isolation of small EVs compatible with exosomes, using UF in combination with SEC. The different MWCOs of the UF devices did not appear to have any impact on the isolated EVs. Our results show that each cell line is different both in the amount of EVs produced and in vesicle protein content. However, the new combination of methods is reproducible independent of cell line employed. This is also supported by the work of Benedikter *et al*. (2017), that furthermore, indicate that this approach tends to be more fitting than the classic UC method [51]. We summarize that isolation and purification of EVs from cell culture supernatants by UF in combination with SEC is a practical, efficient and robust strategy.

## Supporting information

S1 Text Preparation of 30 ml SEC columns. (PDF)

S1 Fig Protein quantification curves from E10, BxPC3, and H3 SEC fractions. Average protein quantification of the size-exclusion chromatography (SEC) fractions. Curves indicate EV fractions from the E10 (A), BxPC3 (B), and H3 (C) cell lines (n = 5) and solely culture media (Advanced DMEM; D and Advanced RPMI; E) (n = 3), both supplemented with 1% exosome depleted FBS. Samples were concentrated prior to SEC in ultrafiltration devices with different molecular weight cut-off (30 kDa, 50 kDa and 100 kDa). Protein quantification (mg/ml) was determined by spectrophotometry (Absorbance 280nm). For the cell culture supernatants, a peak was observed beginning in fraction 9 for the H3 cell line, and in fraction 10 for the E10 and the BxPC3 cell lines. The protein amount in the early protein enriched fractions was similar for the E10 and the H3 cell lines, with the BxPC3 having the lowest values. No significant variation in protein amount in these early EV-enriched fractions was noted between the different molecular weight cut-offs of the ultrafiltration devices within the same cell line (one-way ANOVA using GraphPad Prism, GraphPad Software Inc., version 7.04). The early protein enriched peak was not observed in the culture media (blanks).
(TIFF)
