# Supplementary material for: Efficient extracellular vesicle isolation by combining cell media modifications, ultrafiltration, and size-exclusion chromatography
Source: PLoS One. 2018 Sep 27;13(9):e0204276. doi: 10.1371/journal.pone.0204276 (PMC6160036; doi:10.1371/journal.pone.0204276)
Supplement: S1 Text — (PDF) [file pone.0204276.s001.pdf]

S1 Text. Preparation of 30 ml SEC columns.

Sepharose CL-2B (GE Healthcare Bio-Sciences AB, Uppsala, Sweden) was allowed to equilibrate at room temperature. The sediment was stirred, 150 ml of sepharose was transferred to a beaker and allowed to settle for 60 min and the ethanol supernatant was removed. The sepharose was washed by adding 45 ml of preparation buffer (0.05% sodium-azide in PBS drained through a 0.1 μm filter) through gentle mixing. The sepharose was allowed to settle for 30 min and the supernatant was removed. This washing step was carried out twice. After the last washing step, the supernatant was discarded and 30 ml of preparation buffer was added.

A TELOS™ SPE filtration column 70 ml (900-0000-027T, Kinesis) was placed in a holding rack and leveled before 30 ml of preparation buffer was added. The volume was noted by making a mark on the outside of the column. Next, the column was degassed by pressing a 50 ml syringe plunger into the top of the column until the buffer began to elute. Once the buffer was completely eluted, the column was filled with 60 ml of sepharose, and allowed to settle. Subsequently, a combi-stopper (B. Braun Melsungen AG) was placed on the column tip. Additionally, a TELOS™ 20 μm polyethylene frit (810-0000-027T, Kinesis) was placed rough side up to the bottom of a 50 ml syringe. Then, 10 ml of preparation buffer was added and the frit was degassed with by pressing the plunger into the syringe until the buffer began to elute. The frit was then removed from the syringe, carefully placed on the column (rough side up), and pressed down to the volume marking.
