# Supplementary figures and images for: Efficient extracellular vesicle isolation by combining cell media modifications, ultrafiltration, and size-exclusion chromatography
Source: PLoS One. 2018 Sep 27;13(9):e0204276. doi: 10.1371/journal.pone.0204276 (PMC6160036; doi:10.1371/journal.pone.0204276)

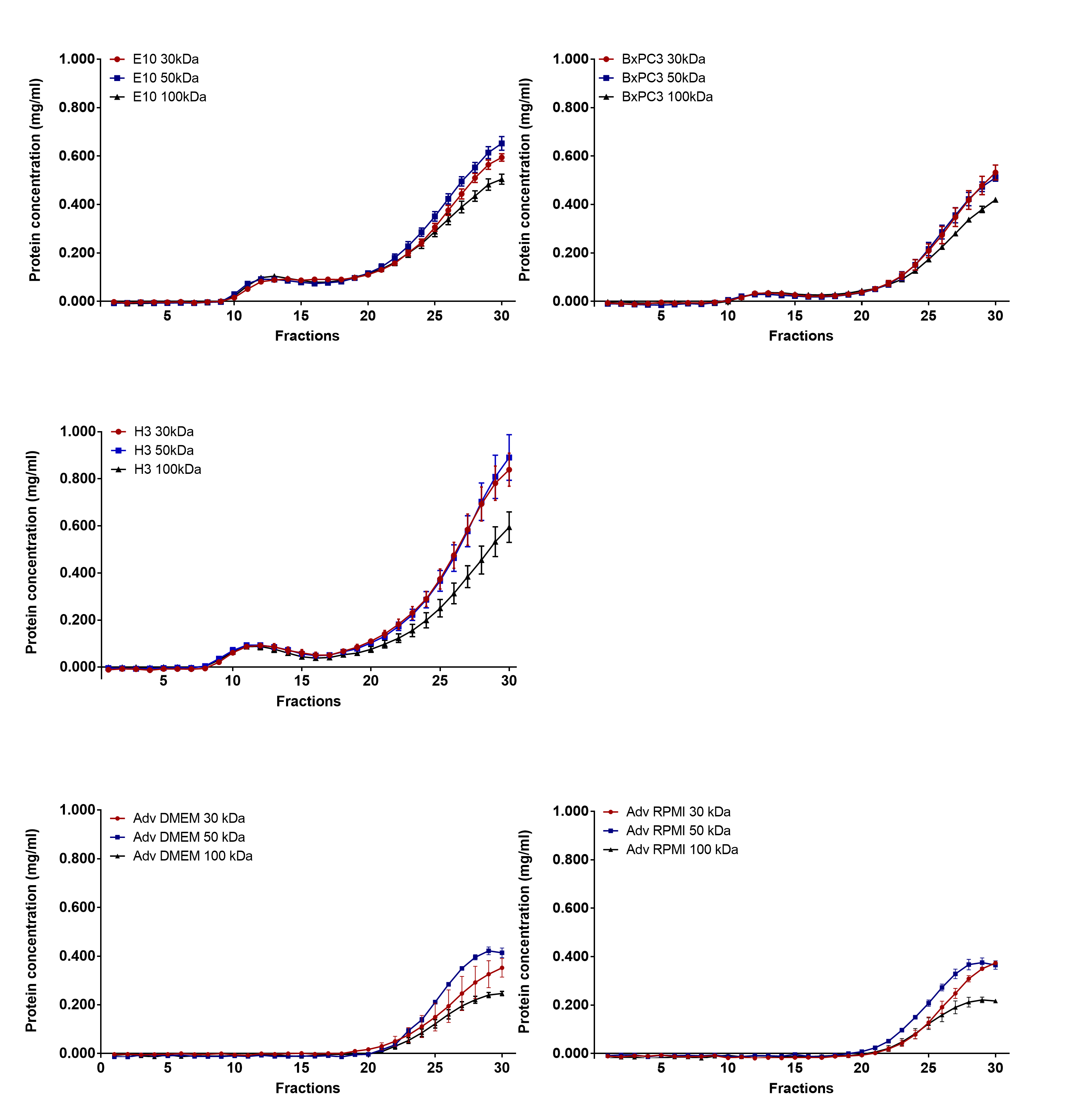

Supplement: S1 Fig — Average protein quantification of the size-exclusion chromatography (SEC) fractions. Curves indicate EV fractions from the E10 (A), BxPC3 (B), and H3 (C) cell lines (n = 5) and solely culture media (Advanced DMEM; D and Advanced RPMI; E) (n = 3), both supplemented with 1% exosome depleted FBS. Samples were concentrated prior to SEC in ultrafiltration devices with different molecular weight cut-off (30 kDa, 50 kDa and 100 kDa). Protein quantification (mg/ml) was determined by spectrophotometry (Absorbance 280nm). For the cell culture supernatants, a peak was observed beginning in fraction 9 for the H3 cell line, and in fraction 10 for the E10 and the BxPC3 cell lines. The protein amount in the early protein enriched fractions was similar for the E10 and the H3 cell lines, with the BxPC3 having the lowest values. No significant variation in protein amount in these early EV-enriched fractions was noted between the different molecular weight cut-offs of the ultrafiltration devices within the same cell line (one-way ANOVA using GraphPad Prism, GraphPad Software Inc., version 7.04). The early protein enriched peak was not observed in the culture media (blanks). (TIFF) [file pone.0204276.s002.tiff]
